# Efficacy and safety of a mammalian target of rapamycin inhibitor in pediatric patients with tuberous sclerosis complex: A systematic review and meta-analysis

**DOI:** 10.3892/etm.2014.2093

**Published:** 2014-11-27

**Authors:** GUANG YANG, LU YANG, XIAOFAN YANG, XIUYU SHI, JING WANG, YUJIE LIU, JUN JU, LIPING ZOU

**Affiliations:** 1 Department of Pediatrics, Chinese PLA General Hospital, Beijing 100853, P.R. China; 2 Special Care Medical Center, Navy General Hospital of PLA, Beijing 100048, P.R. China; 3 Beijing Institute for Brain Disorders, Beijing 100069, P.R. China

**Keywords:** tuberous sclerosis complex, mammalian target of rapamycin inhibitor, pediatric, meta-analysis

## Abstract

Inhibitors of mammalian target of rapamycin (mTOR) are increasingly used as therapy for pediatric patients with tuberous sclerosis complex (TSC). The uncertainty over the efficacy and safety of mTOR inhibitor therapy for the treatment of pediatric patients with TSC emphasizes the necessity for a synthesis of existing evidence. The aim of this study was to assess the efficacy and safety of mTOR inhibitor therapy for the treatment of pediatric patients with TSC. The PubMed, EmBase and Cochrane Library electronic databases were searched, and studies of mTOR inhibitor therapy and non-mTOR inhibitor therapy in pediatric patients with TSC (<18 years old) were selected. Eleven studies met the inclusion criteria. There was evidence of a significantly increased response rate in pediatric patients with TSC treated with mTOR inhibitor therapy compared with those treated with non-mTOR inhibitor therapy (odds ratio, 24.71; 95% confidence interval, 7.46–81.72; P<0.001). The majority of studies reported few adverse events. There was an increased incidence of mouth ulceration, stomatitis, convulsion and pyrexia in pediatric patients with TSC treated with mTOR inhibitor therapy. In conclusion, mTOR inhibitor therapy is an efficacious and safe treatment for pediatric patients with TSC.

## Introduction

Tuberous sclerosis complex (TSC) is a common genetic disorder that results in aberrant cellular differentiation, proliferation and migration early in life (1). TSC occurs in one out of 6,000 newborns and has affected an estimated 1 million individuals globally (2). The condition is characterized by the development of benign tumors in numerous organs (1,3), including the skin (facial angiofibromas), kidney (angiomyolipomas and cysts), lung (lymphangioleiomyomatosis), brain (subependymal giant cell astrocytomas and epileptogenic tuber), heart (rhabdomyomas) and retina (optic nerve tumor) (4–7). These tumors all have the potential to severely affect organ function.

Recent research has revealed the pathogenic mechanism underlying TSC. Mutations in either *TSC1* or *TSC2*, which encode hamartin and tuberin, respectively, cause the abnormal activation of mammalian target of rapamycin (mTOR) (8,9). Hyperactivation of the mTOR pathway, leading to increased cell growth and proliferation, stimulates tumor growth in the brain and other organs in patients with TSC. There are increasing numbers of studies documenting the use of mTOR inhibitors, such as rapamycin, for the treatment of patients with TSC (10,11). These mTOR inhibitors are potentially promising for the treatment of multiple TSC-related tumor types, including renal angiomyolipomas, subependymal giant cell astrocytomas and lymphangioleiomyomatosis (12–18).

The efficacy and safety of mTOR inhibitor therapy in pediatric patients with TSC remain unclear, particularly as a limited response to mTOR inhibitor therapy and drug-related adverse reactions have been reported (19). Given the uncertainty over the treatment effects of mTOR inhibitor and the difficulties in the interpretation of the clinical studies, we therefore carried out a systematic review to assess the efficacy and safety of mTOR inhibitor in the treatment of children with TSC.

## Materials and methods

### Inclusion criteria

This review was reported according to the Preferred Reporting Items for Systematic Reviews and Meta-Analysis statement issued in 2009 (Checklist S1) (20). Quasi-randomized controlled trials (RCTs), case series or case reports comparing any mTOR inhibitor therapy versus placebo or any pretreatment status were included. Studies that were not published as full reports, such as conference abstracts and letters to editors, were excluded. Outcome measures were evaluated by response rates and the incidence of adverse events.

### Search methods for study identification

The PubMed, EmBase and Cochrane Library databases were systematically searched from database inception to July 2013. The search included the following terms: mTOR inhibitor OR rapamycin OR everolimus OR sirolimus AND tuberous sclerosis. In addition, the reference lists of the identified reports, reviews and other relevant publications were manually searched to find other pertinent studies. The medical subject heading, methods, population, study design, intervention and outcome variables of these articles were used to identify relevant studies.

### Data collection and analysis

Two review authors independently examined the titles and abstracts to select eligible studies, and the full texts of the potentially relevant studies were retrieved. Two review authors then independently extracted information from the eligible studies. Data included the first author of the study, sample size, gender, age, disease status, interventions, duration of the follow-up periods, treatment outcomes and adverse reactions. Disagreements concerning study inclusion were resolved through consensus and group discussion.

### Assessment of heterogeneity

Clinical heterogeneity between included studies was assessed by comparing the distribution of important participant factors (e.g. age and gender) between studies and study factors (e.g. loss to follow-up and treatment regimens). Heterogeneity was assessed using the χ^2^ test and I^2^ statistic (21). The χ^2^ test value was interpreted as significant when P<0.1. The I^2^ statistic was interpreted as recommended by Higgins and Green (22): 0–40%, heterogeneity may not be important; 30–60%, heterogeneity may be moderate; 50–90%, heterogeneity may be substantial; and 75–100%, considerable heterogeneity (Higgins 2011). If substantial or considerable heterogeneity was present i.e. P<0.1, I^2^≥50%, the origin of the heterogeneity was evaluated.

### Statistical analysis

A sensitivity analysis was performed to explore the impact of excluding outlying results. Treatment effects were obtained from the number of events reported in each group. The Mantel-Haenszel method was used to evaluate the treatment effect (23,24). Dichotomous data were synthesized using risk ratios. A P-value of 0.05 was used as the cut-off value to determine statistical significance, and data are presented as the estimated effect with 95% confidence intervals (CIs). All statistical analyses were calculated using STATA software (version 12.0; Stata Corp. LP, College Station, TX, USA).

## Results

### Study characteristics

**Figure 1:**
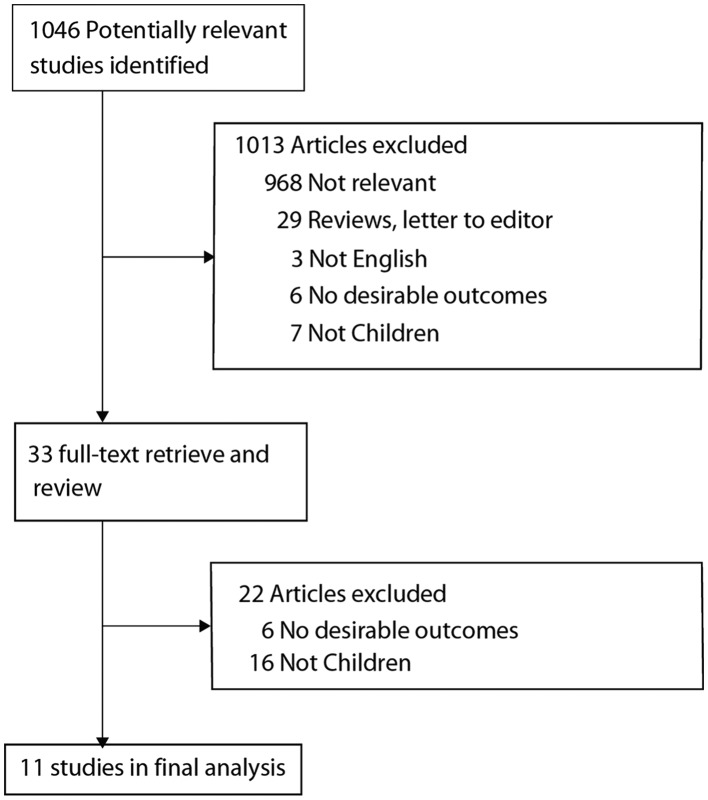
Flow chart of study screening and selection process. A total of 1,046 articles were identified by the initial searches of the medical literature, and 33 required further assessment. Eleven studies were ultimately included in this review.

The characteristics of the included studies (13–19,25–28) are listed in Table I. The initial search retrieved a total of 1,046 potentially relevant publications. The titles and abstracts of the studies were screened and 33 were found to be potentially eligible for inclusion. The full text articles of these 33 studies were retrieved. Subsequent to reading the full texts, 11 studies were found eligible for inclusion according to the criteria for acceptable studies for this review (Fig. 1). The eligible studies reported outcomes on a total of 129 pediatric patients with TSC (<18 years of age). The majority of cases (n=96, 74.4%) received mTOR inhibitor therapy, while the remaining cases (n=33, 25.6%) were treated with non-mTOR inhibitor therapy (Table I). The follow-up for the patients who received mTOR inhibitor ranged between 3.0 and 16.0 months. Ten of the included studies (13–19,26–28) were case series or case reports; the remaining study was an RCT (24). Of the 33 clinical studies that were relevant to mTOR inhibitor therapy in the patients with TSC, 22 were excluded: 16 studies were excluded as they did not include pediatric patients and six were excluded as they did not include outcomes of interest.

### Efficacy of the mTOR inhibitor

**Figure 2:**
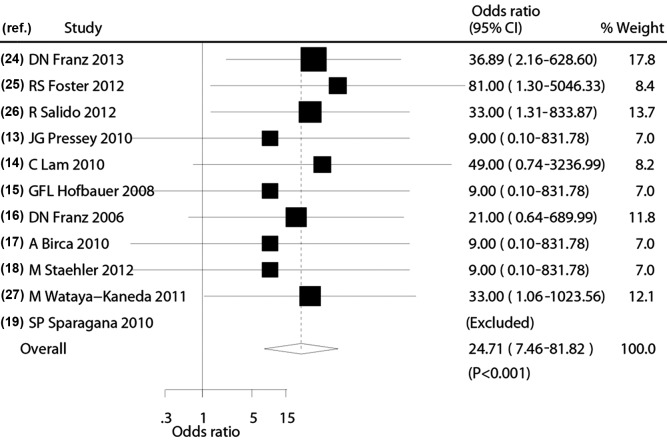
Meta-analysis of clinical response of mTOR inhibitor versus non-mTOR inhibitor therapy. The risk ratio was 24.71 (95% CI, 7.46–81.72; P<0.001). There was no evidence of significant heterogeneity between trials (P=0.13, I^2^=32%). mTOR, mammalian target of rapamycin; CI, confidence interval.

Data reporting clinical response rates subsequent to mTOR inhibitor therapy in pediatric patients with TSC were available from 11 studies (n=129) (13–19,25–28). The meta-analysis demonstrated a significantly increased response rate in pediatric patients with TSC treated with mTOR inhibitor therapy compared with those treated with non-mTOR inhibitor therapy (odds ratio, 24.71; 95% CI, 7.46–81.72; P<0.001; Fig. 2). There was no evidence of significant heterogeneity between trials (P=0.13, I^2^=32%). Sensitivity analysis showed that the results were not affected by the exclusion of any individual study.

### Incidence of adverse events

Data reporting adverse effects associated with mTOR inhibitor therapy and non-mTOR inhibitor therapy for the treatment of pediatric patients with TSC were published in 11 studies (n=129) (13–19,25–28). Pediatric patients that received mTOR inhibitor therapy were more likely to experience mouth ulceration, stomatitis, convulsion, acneiform rash, arthralgias, diarrhea, thrombocytopenia, hyperlipidemia and lipoproteinemia than those treated with non-mTOR inhibitor therapy. The majority of the adverse events were grade 1 or 2 and self-limiting, but some required dose reduction or temporary cessation. The grade 3 adverse events that occurred most frequently were stomatitis, pyrexia and convulsion; grade 4 events were rare (25–28). Non-specific adverse reactions were reported in the treatment group in one study (25). Statistical analysis of the adverse event data was not performed, as the majority of the studies reported few adverse effects.

## Discussion

TSC is a genetic disease affecting multiple systems that causes non-malignant tumors in a number of vital organs, such as the brain, kidneys, heart, eyes, lungs and skin. Numerous symptoms are associated with the condition, including seizures, developmental delay, behavioral problems, skin abnormalities and lung and kidney disease (3). In healthy individuals, *TSC1* and *TSC2* encode hamartin and tuberin, and form the hamartin-tuberin tumor suppressor complex. This inhibits the activation of the mTOR complex 1 (mTORC1), a kinase that modulates protein synthesis and cell growth and proliferation (29,30). In most patients with TSC, a mutation in either *TSC1* or *TSC2* results in an aberrant activation of mTORC1, causing benign tumor growth (31).

The benefit of mTOR inhibitor therapy for pediatric patients with TSC has long been known. Previous studies have shown that rapamycin plays a beneficial role in the treatment of TSC in a mouse model (32,33). A case series demonstrated that rapamycin therapy induced regression of TSC-related astrocytomas and offered an alternative to surgical therapy for these lesions (16). However, one case report (19) indicated that a TSC-related optic nerve tumor was not responsive to rapamycin.

Although nearly all studies conclude that mTOR inhibitor therapy is an effective treatment for TSC, most documented literature is in the form of case studies without any statistical analysis. To summarize the literature and provide preliminary evidence-based treatment guidelines for pediatricians and neurologists, we performed a comprehensive literature search and examined the efficacy of mTOR inhibitor therapy and the possible adverse effects in 129 pediatric patients with TSC. The results of the study suggest that mTOR inhibitor therapy can increase clinical response rates compared with non-mTOR inhibitor therapy. This is the first systematic review investigating the efficacy and safety of mTOR therapy for the treatment of pediatric patients with TSC. Our findings are in agreement with a recently published RCT (25).

Several mechanisms for the antitumor effects of mTOR inhibitors have been proposed. Firstly, mTOR inhibitors have been suggested to inhibit mTOR-regulated processes by reducing the phosphorylation of downstream mTOR effectors, including the translational repressor eukaryotic elongation factor 4E binding protein 1 and the S6 ribosomal protein kinase 1. This rehabilitates the translation of pivotal proteins involved in cell cycle regulation, glycolytic activity, angiogenesis, cell size control and cellular growth (34,35). Secondly, mTOR inhibitors reduce the expression of angiogenic factors, such as vascular endothelial growth factor (VEGF). VEGF can promote neovascularization, which plays a significant role in the development of solid tumors (36,37).

The most common adverse events in pediatric patients with TSC treated with mTOR inhibitor therapy were mouth ulceration, stomatitis, convulsion and pyrexia (25). The majority of the adverse events were grade 1 or 2 and self-limiting, but some required dose reduction or temporary interruption of treatment. The most common grade 3 adverse events were stomatitis, pyrexia and convulsion (25–27). Infection in the upper respiratory tract was also reported. It is noteworthy that a 17-year-old girl experienced secondary amenorrhea, which may have been a consequence of mTOR inhibitor therapy as previous data suggest that mTOR can suppress puberty onset (38).

There were several limitations to this study. Firstly, in accordance with the inherent assumptions made when performing any meta-analysis, this study was based on pooled data, which may not provide a detailed relevant analysis. Secondly, different TSC disease status could have influenced our conclusions about the response rates subsequent to mTOR inhibitor therapy. Thirdly, data on any specific adverse event were unavailable in these studies; therefore, the association between any specific type of adverse event and mTOR inhibitor therapy was not identified. The long-term assessment of the potential adverse effects of mTOR inhibitor therapy on growth, development and sexual maturation in the pediatric population remains to be resolved.

Future studies should focus on the efficacy and safety of mTOR inhibitor therapy in combination with other drugs to provide an optimal treatment strategy, as well as the efficacy and safety of mTOR inhibitor therapy for the treatment of specific TSC subtypes.

## Figures and Tables

**Table I:** Characteristics of the 11 studies included in the meta-analysis.

First author, year (ref.)	Sample size (MC/NC)	Gender (M/F)	Age (years)	Inclusion criteria	Intervention^a^	Follow-up (months)
Franz, 2013 (24)	68/33	NR	1.0–18.0	Subependymal giant cell astrocytomas associated with TSC	Everolimus	9.7
Foster, 2012 (25)	4/0	2/2	5.0–17.0	Facial angiofibromas associated with TSC	Sirolimus	6.0
Salido, 2012 (26)	7/0	4/3	6.0–14.0	Facial angiofibromas associated with TSC	Sirolimus	9.0
Sparagana, 2010 (19)	1/0	0/1	12.0	Optic nerve tumor associated with TSC	Sirolimus	16.0
Pressey, 2010 (13)	1/0	1/0	7.0	Fibromatosis and multifocal renal cell carcinoma associated with TSC	Sirolimus	6.0
Lam, 2010 (14)	3/0	2/1	9.0–13.0	Subependymal giant cell astrocytomas associated with TSC	Sirolimus	3.0
Hofbauer, 2008 (15)	1/0	0/1	15.0	Facial angiofibromas associated with TSC	Rapamycin	9.0
Franz, 2006 (16)	4/0	2/2	3.0–15.0	Subependymal giant cell astrocytomas or a pilocytic astrocytoma associated with TSC	Sirolimus	3.0
Birca, 2010 (17)	1/0	0/1	8.0	Subependymal giant cell astrocytomas associated with TSC	Rapamycin	3.0
Staehler, 2012 (18)	1/0	0/1	12.0	Renal angiomyolipoma associated with TSC	Sirolimus	6.0
Wataya-Kaneda, 2011 (27)	5/0	2/3	9.0–17.0	Facial angiofibromas associated with TSC	Rapamycin	3.0

a Represented by the drug name stated in the study.

MC, cases receiving mTOR inhibitor therapy; NC, cases receiving non-mTOR inhibitor therapy; M/F, male/female; NR, not reported; TSC, tuberous sclerosis complex; mTOR, mammalian target of rapamycin.
